# The Epistemic Uncertainty Gradient in Spaces of Random Projections

**DOI:** 10.3390/e27020144

**Published:** 2025-02-01

**Authors:** Jeffrey F. Queißer, Jun Tani, Jochen J. Steil

**Affiliations:** 1 Cognitive Neurorobotics Research Unit, Okinawa Institute of Science and Technology Graduate University, Onna 904-0495, Japan; jun.tani@oist.jp; 2 Institut für Robotik und Prozessinformatik, Technische Universität Braunschweig, 38106 Braunschweig, Germany; j.steil@tu-braunschweig.de; 3 Theoretical Sciences Visiting Program (TSVP), Okinawa Institute of Science and Technology Graduate University, Onna 904-0495, Japan

**Keywords:** associative memory, probabilistic, epistemic uncertainty, unlearning, one shot, iterative, regression

## Abstract

This work presents a novel approach to handling epistemic uncertainty estimates with motivation from Bayesian linear regression. We propose treating the model-dependent variance in the predictive distribution—commonly associated with epistemic uncertainty—as a model for the underlying data distribution. Using high-dimensional random feature transformations, this approach allows for a computationally efficient, parameter-free representation of arbitrary data distributions. This allows assessing whether a query point lies within the distribution, which can also provide insights into outlier detection and generalization tasks. Furthermore, given an initial input, minimizing the uncertainty using gradient descent offers a new method of querying data points that are close to the initial input and belong to the distribution resembling the training data, much like auto-completion in associative networks. We extend the proposed method to applications such as local Gaussian approximations, input–output regression, and even a mechanism for unlearning of data. This reinterpretation of uncertainty, alongside the geometric insights it provides, offers an innovative and novel framework for addressing classical machine learning challenges.

## 1. Introduction

Machine learning, as an inductive methodology that creates models from data, is fundamentally about uncertainty, particularly when making predictions based on these models. Modern machine learning often uses probabilities to express the associated uncertainties. However, as Hüllermeier and Waegeman [1] pointed out in a recent review: “In particular, this includes the importance of distinguishing between (at least) two different types of uncertainty, often referred to as aleatoric and epistemic.” (p. 457). The former refers to the irreducible uncertainty introduced by the randomness of the modeled data, while the latter commonly refers to the uncertainty associated with the lack of knowledge about model parameters for a given class of models and can, in principle, be reduced by adding more data. Providing a historical perspective, Hacking [2] states that the academic discourse on aleatoric and epistemic uncertainty dates back to the seventeenth century, while older work on probabilities addresses the distinction between aleatoric and epistemic uncertainty [3,4].

Until recently, the topic of different types of uncertainty has rarely been considered in machine learning; among the first to motivate considering it in a machine learning context were Senge et al. [5], while more recently there has been an uptick of interest in the topic, i.e., in the context of privacy or in variational approaches [6,7]. But even adopting common notions of aleatoric vs. epistemic uncertainty is not generally agreed upon, nor is it easy to quantify them [1]. Furthermore, as the authors Hüllermeier and Waegeman [1] emphasize, “What this example shows is that aleatoric and epistemic uncertainty should not be seen as absolute notions. […] Changing the context will also change the sources of uncertainty: aleatoric may turn into epistemic uncertainty and vice versa.” ([1], p. 464).

Particularly well understood is uncertainty in Bayesian regression, both for concept learning and numerical regression of parameterized models, where a concept typically is associated with a specific model parameter vector. Given the model class, the model parameters are averaged out to form the Bayesian predictive distribution, and the resulting total uncertainty is known to mix aleatoric and epistemic components ([8], p. 156). These can be identified in the variance of the predictive distribution. We follow the Bayesian regression approach and employ as specific feature space a dedicated fixed high-dimensional random feature transformation that is well known to yield universal approximation capabilities as well as very efficient computation when used in the context of functional-link networks [9] or the so-called extreme learning machines [10]. But here, we are not interested in the well-known prediction abilities of this random feature model.

**Figure 1:**
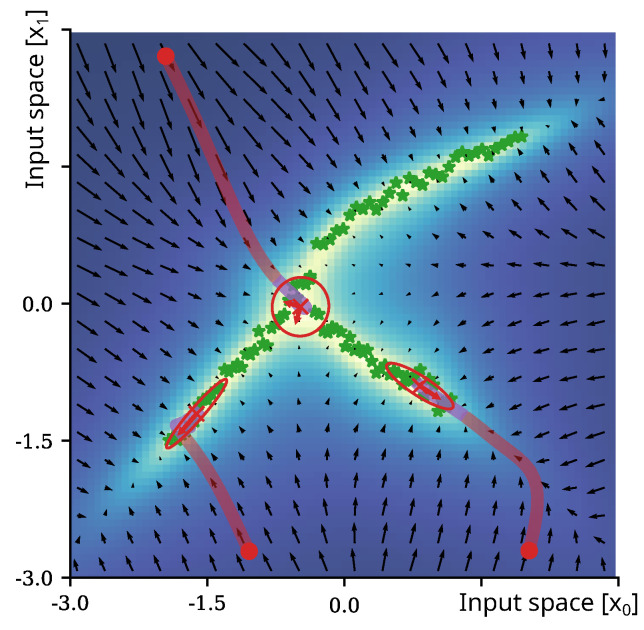
Visualization of the epistemic uncertainty of an exemplar 2D complex (non-normal) distribution. The green stars depict samples; the colored area and vector field indicate the epistemic uncertainty in the input space and its gradient, respectively. For the three initial inputs, the trajectory during minimization of the epistemic uncertainty through gradient descent over 50 iterations is shown. The red sections of the trajectories indicate states that are classified as outside of the learned distribution, while the purple sections indicate iterations towards areas of high certainty that are already classified as belonging to the distribution of the training data. The oval annotations depict estimated local Gaussian approximations at converged solutions.

This paper, rather, introduces a novel concept to treat the model-dependent part of the variance of the predictive distribution ([8], p. 156), which is commonly denoted “epistemic uncertainty” in this Bayesian linear regression setting [4,11], as a model of the underlying distribution of the data. We have adopted this notion (Figure 1), although it does not fully separate epistemic and aleatoric uncertainty, as can be easily seen from its analytical computation (see Section 2). This epistemic uncertainty results from averaging over the possible parameters weighted by their posteriors. It depends solely on the given data and the feature transformation and, thus, is a parameter-free and easy-to-compute model. It is also worth noting that it is the diagonal of the so-called smoothing kernel ([8], chap. 3.3.3) and is also closely related to the Mahalanobis distance for centered data, as detailed in Section 2.2.

We argue that under the given high-dimensional random feature transformation and for a specific input query point the variance of the predictive output distribution gives a good measure of whether this query point is “in-distribution” or not. In this vein, our method is conceptually related to others that try to approximate the support of a distribution to determine if a given query point is in or out of distribution, e.g., DeVries and Taylor [12], Lee et al. [13], Malinin and Gales [14], Sensoy et al. [15]. Such approaches, however, often rely on parametric models such as Gaussian mixtures, path length estimations in binary trees (e.g., the isolation forest algorithm), or computationally expensive approximations of the underlying data distribution via deep neural networks, which can introduce other intricate issues. Our method shares with these models the concept of thresholding the epistemic uncertainty when determining whether a data point belongs to the in- or out-of-distribution class.

A further source of inspiration comes from the following question: how close is a given query point to a distribution or what is the nearest in-distribution point? For centered distributions with mean zero, one possible answer is to compute the Mahalanobis distance, which gives the variance-weighted distance to the mean. This, however, does not answer the question of what could be the closest point in a distribution relative to a query. The evaluation of the epistemic uncertainty provides us with a constructive method to determine one. Its gradient can be computed analytically, and, therefore, an iterative gradient descent can be devised that minimizes the epistemic uncertainty until the threshold to “in-distribution” is hit, to find the “closest point”. Note that this neither provides a proper metric, nor does it relate to the global statistics of the full dataset like the Mahalanobis approach. Rather, we define the gradient field of the epistemic gradient and follow it until the prediction variance becomes small, which is possible for all types of data distributions. To the best of our knowledge, this is an entirely new method.

Despite its simple derivation and computation, we further show that following the epistemic gradient has a very interesting and novel geometrical interpretation that uses specific properties of high-dimensional random feature transformation. Interestingly, the theoretical analysis shows that training data are not represented as attractors in this feature space. In contrast to many common approaches that are based on direct data representation (e.g., [16,17,18,19]), here, the dynamics towards the data distribution are generated by weighted repulsive forces in the hidden space. Note that this interpretation resembles, to some degree, the interpretation of linear regression with recurrent random features in [20,21,22,23], which, however, was derived in a very different context. We show that this concept of *avoidance of the unknown*, rather than the usual *convergence to the known*, provides an innovative method for data representation and can be beneficial for generalization and extrapolation. Through extensions of the method, we further show that local Gaussian approximations of the data distribution can be computed, that ordinary but very flexible input–output regression is possible in an auto-associative mode, that outliers and anomalies can be detected and benchmarked favorably against standard methods, and, finally, we devise a constructive method for unlearning of data.

In summary, inspired by the recent discussion on aleatoric vs. epistemic uncertainty in machine learning, we reinterpret a well-known term in Bayesian linear regression that determines the variance of the predictive distribution as a feature-transformation-dependent model of the data distribution, which signifies a kind of “epistemic uncertainty distribution model”. Through the choice of the specific high-dimensional random feature transformation, we obtain a geometric interpretation of the respective uncertainty gradient, which itself is a new concept. We then show that through this new method, a number of classical problems can be tackled, including regression and outlier detection, whereas we do not have to make any assumptions on the original data distribution.

## 2. Preliminaries

### 2.1. The Epistemic Uncertainty

Considering Bayesian linear regression (e.g., [8]) of a distribution of target values y, the latter is conditioned on weights W and features $x_{\phi }=\phi \left(x\right)$ for inputs x, for some feature space mapping ϕ(x) and Gaussian data noise modeled with precision β:

(1) $$p\left(y|x_{\phi },W,\beta \right)=N\left(y|x_{\phi }W,\beta^{-1}\right).$$

Using the design matrix $X_{\phi }$, the posterior distribution of the output weights W is given as

(2) $$p\left(W|X_{\phi },Y,\alpha ,\beta \right)=N\left(W|m_{N},S_{N}\right).$$

Under the assumption of Gaussian prior $S_{0}=\alpha^{-1}I$ and sample precision $\beta =\sigma_{x}^{-1}$, the posterior of weights is parameterized, such that

(3) $$\begin{array}{cc} m_{N} & =\beta S_{N}X_{\phi }Y,\;\mathrm{and}\; \end{array}$$

(4) $$\;S_{N}^{-1}=\alpha I+\beta X_{\phi }^{T}X_{\phi }.$$

Integrating out W, the full Bayesian predictive distribution of outputs y is given by parameterized distribution, such that

(5) $$p\left(y|x_{\phi },X_{\phi },Y,\alpha ,\beta \right)=N\left(y|m_{N}^{\;T}x_{\phi },\sigma_{N}^{2}\left(x_{\phi }\right)\right).$$

The variance of the predictive distribution for a provided input $x_{\phi }$ is decomposed into aleatoric and epistemic uncertainty as

(6) σN2(xϕ)=xϕTβ−1︸(a)aleatoric+xϕTSNxϕ︸(b)epistemic.

The aleatoric uncertainty is irreducible and originates in the data noise, i.e., the output *y* is modeled as a Gaussian Equation (1) capturing the variance of the training dataset. The epistemic uncertainty $U\left(x\right)=x_{\phi }^{\;T}S_{N}x_{\phi }$ is the uncertainty related to the model parameters W, which decreases with the increasing number of training samples. As discussed above, this common definition of the epistemic uncertainty in the literature captures the dependency on parameterization—that is, the epistemic part, but as the definition of $S_{N}$ Equation (4) still depends on β it does not fully separate epistemic and aleatoric uncertainty. As this does not impede our further proceeding, we simply follow the common notion.

### 2.2. The Mahalanobis Distance

For dataset X with mean μ the Mahalanobis distance of a point x is defined as

(7) $$\begin{array}{c} D_{M}={\left(x-\mu \right)}^{\;T}\Sigma^{-1}\left(x-\mu \right). \end{array}$$

$D_{M}$ measures the distance of x to the mean μ of *X* in terms of standard deviations of the dataset X, where $\Sigma^{-1}$ is the covariance matrix.

Let x∈X be a vector and $X=[x_{0},\cdots ,x_{n}]$ be some centered example data with mean μ=0. Now, perform linear regression directly in the input space X; then, the epistemic uncertainty derived above Equation (6) is identical to the Mahalanobis distance, since the covariance matrix $\Sigma =X^{\;T}X$ equals the product of the design matrix (in this case, this equals the transpose of the data matrix) with its transpose. This shows that, in principle, the epistemic uncertainty is related to the Mahalanobis distance. It does measure something like the distance to a given distribution, which is represented by a sample dataset.

However, in this baseline case, minimization of epistemic uncertainty with respect to some data point x is not helpful, because the minimum will always be the mean, by definition. However, this changes if we consider an intermediate feature transformation of x into some feature space $X_{\phi }$, as introduced next.

## 3. Method

### 3.1. The Feature Space Transformation

In the following, we consider as feature transformation $x\mapsto \phi \left(x\right)\in X_{\phi }$ with $x\in R^{d}$ and $\phi \left(x\right)\in R^{k}$, the well-known random feature neural network with fixed random input weights and standard sigmoidal non-linear activation function [24,25,26]:

(8) $$\phi (x)=f_{\sigma }\left(W_{inp}x+b\right),\;\mathrm{and}$$

(9) $$\;f_{\sigma }\left(x\right)=\frac{1}{1+e^{-x}},$$

where $W_{inp}\in R^{k\times d}$ is a random matrix with entries drawn according to $w_{ij}\;\;∼\;\;N\left(0,\sigma \right)$, $b\;\in \;R^{d}$ is an optional bias term defined likewise as $b_{j}∼\;N\left(0,\sigma \right)$, and $f_{\sigma }$ is applied component-wise. Note that the feature dimension *k* is supposed to be much larger than the input dimension *d*: k≫d, so that the feature transform performs a strong upscaling. However, as Equation (8) is locally a diffeomorphism, the feature mapping creates a local *d*-dimensional submanifold in the *k*-dimensional feature space. Furthermore, due to the random summation and for large *k* the norm of the data in the feature space is constant $\left|\right|x_{\phi }\left|\right|\approx C$ for some value of C, dependent on the feature dimension and the form of the sigmoidal activation function.

Geometrically speaking, after feature transformation the data $X_{\phi }$ is located on a *d*-dimensional sphere in feature space $X_{\phi }$, which will be of the utmost importance to interpreting the gradient of the epistemic uncertainty defined below. Moreover, the projection in the feature space is restricted to the non-negative orthant of the manifold, because $f_{\sigma }$ is defined as a logistic activation function. This does not impede the representations of arbitrary distributions, as discussed in the following by means of a geometric interpretation of the feature space.

### 3.2. The Epistemic Gradient

We first derive the gradient of the epistemic uncertainty, introduced as part of Equation (6), with respect to an input space vector, the input x. The basic idea is to treat $U\left(x\right)=x_{\phi }^{\;T}S_{N}x_{\phi }$ as an objective function (the uncertainty) to be minimized with respect to the data space input x, where $x_{\phi }=\phi \left(x\right)$ is a shortcut notation for the feature space vector. Given

(10) $$\begin{array}{ccc} \nabla_{x}U & =\frac{\partial f(z)}{\partial z}\cdot \frac{\partial z}{\partial x},\; & \mathrm{with}\\ z & =\phi (x),\; & \mathrm{and}\\ f(z) & =z^{\;T}S_{N}z, \end{array}$$

in case of sigmoidal activation functions, the derivative of *z* is given by $\frac{\partial z}{\partial x}=\left[\phi \left(x\right)\left(1-\phi (x)\right)\right]W_{inp}^{\;T}$ and $\frac{\partial f(z)}{\partial z}$ as

(11) $$\begin{array}{cc} \frac{\partial f(z)}{\partial z_{i}} & =\frac{\partial z^{\;T}S_{N}z}{\partial z_{i}}=\frac{\partial }{\partial z_{i}}\sum_{j,k}S_{N_{j,k}}z_{j}z_{k}\\ \; & =\frac{\partial }{\partial z_{i}}\left[\sum_{j\neq i,k\neq i}S_{N_{j,k}}z_{j}z_{k}+\sum_{j\neq i}S_{N_{j,i}}z_{j}z_{i}+\sum_{k\neq i}S_{N_{i,k}}z_{i}z_{k}+S_{N_{i,i}}z_{i}^{2}\right]\\ \; & =\sum_{j\neq i}S_{N_{i,j}}z_{j}+\sum_{k\neq i}S_{N_{i,k}}z_{k}+2S_{N_{i,i}}z_{i}\\ \; & =2\sum_{j}S_{N_{i,j}}z_{j}. \end{array}$$

Finally, the gradient of U with respect to x is defined as

(12) $$\begin{array}{cc} \nabla_{x}U & =\frac{\partial f(z)}{\partial z}\frac{\partial z}{\partial x}=2W_{inp}\left[S_{N}\phi \left(x\right)\right]\left(\phi \left(x\right)\left(1-\phi (x)\right)\right). \end{array}$$

### 3.3. Geometric Interpretation

As introduced in Equation (4), the inverse of the posterior’s variance $S_{N}^{-1}$ is a real positive-definite and a symmetric matrix; hence, its spectral decomposition

(13) $$S_{N}^{-1}=Q\Lambda Q^{-1}=Q\Lambda Q^{\;T}=\left[\begin{array}{c} q_{0}\\ \vdots \\ q_{k} \end{array}\right]\left[\begin{array}{ccc} \lambda_{0} & \; & \\ \; & \ddots & \\ \; & \; & \lambda_{k} \end{array}\right]{\left[\begin{array}{c} q_{0}\\ \vdots \\ q_{k} \end{array}\right]}^{\;T}$$

is defined and a computationally efficient inversion through element-wise inversion of eigenvalues $\lambda_{i}$ is possible. The substitution of $S_{N}$ in the epistemic uncertainty $x_{\phi }^{\;T}S_{N}x_{\phi }$ by its spectral decomposition results in

(14) U=xϕTQΛ−1QTxϕ=xϕTQ1λ0⋱1λkQTxϕ,

which can be rearranged into a squared sum of scalar products (cosine similarities) that are weighted by the reciprocals of their respective eigenvectors, such that

(15) U=〈q0,xϕ〉⋮〈qk,xϕ〉1λ0⋱1λk〈q0,xϕ〉⋮〈qk,xϕ〉T=∑i=0k〈qi,xϕ〉2λi=∑i=0kei.

**Figure 2:**
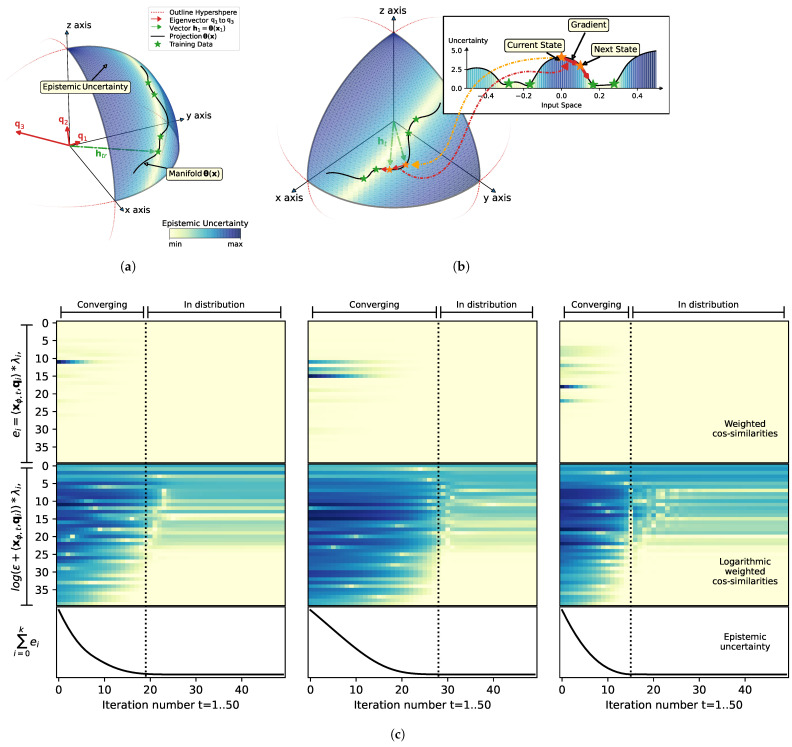
(**a**) Geometrical interpretation of feature space; (**b**) Visualization of minimization of the epistemic uncertainty in the input space. The visualization of the hidden space representation of the proposed method in (**a**,**b**) depicts training data as green stars on the manifold (black line) along a hypersphere in the three-dimensional hidden space. Color shading of the surface of the hypersphere (positive orthant) indicates the epistemic uncertainty. An additional projection of the one-dimensional input into the hidden space is shown in panel (**b**). Optimization (minimization of epistemic uncertainty through gradient) for novel inputs (orange starts) is performed iteratively and indicated by red arrows. Scaling (length) of eigenvectors ($q_{1}$, $q_{2}$, and $q_{3}$) according to function log1λn; (**c**) Internal analysis of the weighted orthogonalization of the current estimate to the eigenvectors of the training data during minimization of the epistemic uncertainty. Minimization of epistemic uncertainty optimization is sown for three random inputs over 50 steps (horizontal axis). Epistemic uncertainty (bottom) is defined as sum of squared covariance similarities between the projected input into the hidden space and the eigenvectors of the training data distribution. The top plot shows magnitude of the cosine similarities (color shading). The middle plot shows the logarithmic transformation of the cosine similarities.

Following from Equation (15), minimizing the epistemic uncertainty can be considered as maximizing the orthogonality of the next estimate $x_{\phi }^{\left(n+1\right)}$ to all eigenvectors $q_{i}$ in the input space. Due to the weighting of the scalar products with the inverse eigenvalues $\lambda_{i}$, orthogonalization to directions that represent the training distribution $X_{\phi }$ the least can be considered a primary objective, as the respective eigenvalues are typically multiple magnitudes larger in comparison to the eigenvectors representing the training distribution. As a result of the minimization of the epistemic uncertainty in the input space of the non-linear mapping ϕ(x), optimization is non-trivial (see discussion in Section 2) and results in convergence towards local minima representing the training data distribution of $X_{\phi }$, as depicted in Figure 2.

Figure 2a,b show the results of a toy example of a 1D input that is projected into a 3D feature space. It can be seen how the training samples, marked by green stars, are projected onto a manifold in the positive orthant of a sphere in the feature space. The training samples are located in regions of minimum uncertainty (indicated by shading of the hypersphere surface), which is due to their maximum weighted orthogonality to all eigenvectors $q_{1}$, $q_{2}$, and $q_{3}$ (highlighted in red). The weighing of the eigenvectors is visualized by the length estimated as the log of the reciprocal of the eigenvalues. Therefore, it can be seen that $q_{1}$ is very small (owing to its largest eigenvalue) and that it represents the mean of the training data in the positive orthant of the hidden space. Due to its small contribution to the final uncertainty estimate, $q_{1}$ is irrelevant and can be ignored. This shows that centering of training data is not necessary as long as the data value range aligns with the slope and the centers of the non-linear activation functions. In fact, orthogonalization to $q_{1}$ cannot even be achieved, since all projections from the input space are restricted to the positive orthant of the hidden space. The second eigenvector, $q_{2}$, represents most of the variance in the training data, as its scaling by its inverse eigenvalue is also small and contributes only insignificantly to the gradient field of the epistemic uncertainty.

The main contributor to the estimate of the epistemic uncertainty in our case is $q_{3}$, which is a strong repeller. As $q_{3}$ represents the least variance of the training samples, as the training samples are almost orthogonal to $q_{3}$, and as the more novel inputs deviate from the observed training samples, the more likely it is that the projection of the samples into the hidden space loses its orthogonality to $q_{3}$. This means that eigenvectors with low eigenvalues can be considered as “novelty detectors”, which cause increased uncertainty estimates in cases where a deviation from the training distribution occurs. During the minimization of the epistemic uncertainty, these novelty detectors can be interpreted as repellers, as current estimates are being pushed away from the respective eigenvectors through optimization via gradient descent.

Note that for visualization purposes, the depicted example only operates in a three-dimensional feature space, and that, due to this limitation, spurious minima can occur, i.e., an increase in orthogonality in the feature space with the further increased distance of samples from the training data distribution. Indeed, in our example case the epistemic uncertainty is decreasing with input space approaching −0.5, i.e., we can identify a spurious minimum. This observation is common in cases of low-dimensional feature spaces and high non-linearity; nevertheless, our empirical analysis shows that with a growing number of (random) hidden features the probability increases that at least one dimension “detects” the deviation from the observed distribution. This means that the orthogonality to one of the eigenvectors that contribute as novelty detectors strongly increases and acts as a strong repeller to force the network states back towards the observed distribution.

In addition, a visualization of the orthogonalization in relation to the minimization of the epistemic uncertainty is shown in Figure 2c. Each subplot shows the contributions of each eigenvector to the overall uncertainty estimation for the three optimizations of Figure 1. The top panel shows the squared weighted cosine similarity $e_{i}$ as introduced in Equation (15), the middle panel shows a log plot of $e_{i}$, and the lower panel shows the epistemic uncertainty U during minimization of the epistemic uncertainty for 50 steps. The visualization of orthogonality to eigenvectors is sorted by the magnitude of the respective eigenvalues. The log plot, in particular, reveals two types of eigenvectors: (1) eigenvectors that do not represent the training data (repellers), whose contributions can be minimized, and (2) eigenvectors that represent the training data, which maintain low orthogonalization with respect to minimized solutions and, as a result, do not contribute to the minimization of epistemic uncertainty. Our empirical analysis (e.g., Figure 2) showed that heuristics $U\left(x\right)<10x_{\min}$ with $x_{\min}=\mathrm{argmin}\left(U(x)\right)$ is a good indicator for deciding when iterative minimization of the epistemic uncertainty reaches the data distribution, as shown by the dashed black lines in Figure 2c. Here, we only considered eigenvectors 1λ>10−6 for the computation of the threshold operations.

### 3.4. Parameterization of Prior

**Figure 3:**
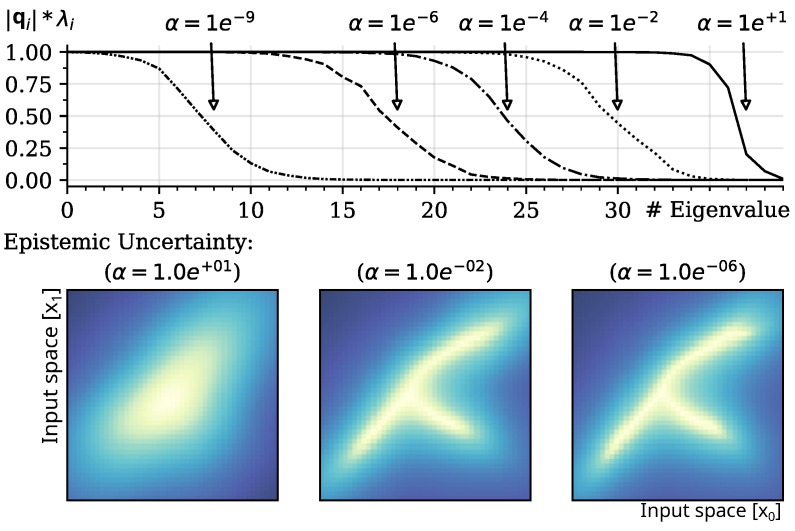
Top panel: Shift of eigenvalue spectrum causes a weighting of cosine similarities in the calculation of the epistemic uncertainty, induced by modulation of variance α of prior $S_{0}\;=\;\alpha^{-1}I$ and with sample precision set to β=1. Bottom Panels: The resulting effect of “regularization” in the representation of training distributions (smoothing of epistemic uncertainty) caused by modulation of variance α, with $\alpha =1\times 10^{1}$, $\alpha =1\times 10^{-2}$, and $\alpha =1\times 10^{-6}$. The results show a mean of n=10 runs of random projections into a k=40-dimensional hidden space. Dark/blue areas indicate lower epistemic uncertainty as projected into the input space.

Prior knowledge in linear regression is usually provided in the form of regularization λreg=αβ and can be interpreted as weighting of an L2 regularization term for estimation of the output weights of the model. Although the proposed method does not estimate output weights, the parameterization influences the representation of internalized samples through the interaction during estimation of epistemic uncertainty with $S_{N}^{-1}$. The effect of changes in the precision of the prior through α is shown in Figure 3.

Due to the definition of $S_{N}^{-1}$, Equation (4), hyperparameter α induces a spectrum shift (shown in Figure 3; top panel) in the eigenvalue spectrum and adjusts the number of vectors considered for orthogonalization during minimization of the epistemic uncertainty. The related Equation (15) shows the inverse relationship of eigenvalues and the weighting of cosine similarities. As a result, the estimation of the epistemic uncertainty gradually depends on a smaller number of representative features and becomes smoother as α increases. Interestingly, the resulting effect is similar to the standard regularization of output weights in regression models; the stronger the regularization is performed, the smoother the learned mapping from input to output.

### 3.5. Method Application

#### 3.5.1. The Auto-Associative Case

Given an initial input of pattern $x^{\left(0\right)}$, the iterative minimization of the epistemic uncertainty of estimates based on the gradient in Equation (12),

(16) $$\begin{array}{cc} x^{\left(n+1\right)} & \leftarrow x^{\left(n\right)}-\eta \nabla_{x^{\left(n\right)}}U, \end{array}$$

results in $x^{\left(n\right)}$, an estimate with maximized epistemic uncertainty and high similarity to the presented training data. The constant η is usually introduced in this case as the update rate for optimization. Figure 1 shows the trajectories of $x^{\left(n\right)}$ for three random initial states $x^{\left(0\right)}$ of a 2D distribution; the epistemic uncertainty is encoded by background coloring, and the gradient of the epistemic uncertainty is visualized as arrows of a vector in black.

#### 3.5.2. The Regression Case

The proposed method creates a nonparametric representation of the joint probability of the dataset in an unsupervised manner. Therefore, in contrast to typical regression models, no mapping is estimated between the feature space and the output data.

Nevertheless, implementation of regression is possible by autocompletion or auto-association of patterns, when treating concatenated inputs and respective outputs as elements of a joint input data space. This approach is common to a number of classical neural network and machine learning methods, including original pattern auto-association in the Amari–Hopfield network [27,28] or by using variations of the self-organizing maps [29,30]. Early autocompletion through recurrent network dynamics has been proposed by [31] in a context of robotics; more recent work addresses multi-dimensional and multi-modal continuous association [32]. In probabilistic modeling, Gaussian Mixture Regression follows a similar idea of first modeling the joint distribution of inputs and outputs and then marginalizing to obtain the desired output for a given input [8]. Our approach is on an intermediate ground. It uses probabilistic modeling of the joint distribution based on a feature transform, but resorts to an iterative minimization procedure rather than to explicit marginalization.

To this end, we consider the input feature space $z={\left[x^{\;T},y^{\;T}\right]}^{\;T}\in R^{d+e}$ as a concatenation of inputs $x\in R^{d}$ with respective outputs $y\in R^{e}$. Given a query input vector $z^{\left(0\right)}={\left[x^{\;T},y^{\left(0\right)\;T}\right]}^{\;T}\in R^{d+e}$ with an initial estimate of output $y^{\left(0\right)}$, an output can be estimated by iterative minimization of the epistemic uncertainty Equation (16) restricted to $y^{\left(n+1\right)}$. The input vector x is considered to be immutable (provided input) and is clamped to its initial value.

This approach requires an iterative update towards a solution, but there are benefits: multiple solutions (ambiguities) can be represented and queried by variations of the initial estimate of $y^{\left(0\right)}$. Solutions that are closer to the initial estimate provided can be assumed to be more likely to be discovered. As an example, consider a typical inverse kinematics task, such as robot reaching. If a desired target moves continuously in space, solutions of consecutive configurations that are more similar to each other would be considered beneficial for smooth and safe operation of the robot.

A second benefit of the presented approach is that there is no structural differentiation between inputs and outputs, as in all auto-associative approaches. It can, thus, operate in inverse operation and estimate the most likely inputs given the desired output vectors. Even more so, each individual dimension of the input space of the method can be considered independently as input or output, or it can be dynamically configured between forward and inverse modes of operation.

### 3.6. Extended Method Applications

#### 3.6.1. Local Gaussian Approximation

In the following, we denote the solution of the iterative minimization of the epistemic uncertainty Equation (16) by gradient descent from an initial state $x^{\left(0\right)}$ as a converged solution $x^{\left(N\right)}$. The number *N* of necessary iterations can vary, and iterative optimization is typically performed until a certain precision criterion is met, such as, for example, $\nabla U_{x^{\left(n\right)}}<\epsilon$. Furthermore, we assume that $x^{\left(N\right)}\approx x^{*}$ is a true local minimum, which implies that the Hessian at this point is positive-definite.

Under the assumption that the uncertainty estimates in a local neighborhood of $x^{*}$ resemble a Gaussian shape, we can consider a local approximation through a multivariate Gaussian probability density function (PDF) with variance $\Sigma_{x^{*}}$ and mean $x^{*}$ in a *k*–dimensional space:

(17) $$p\left(x|\Sigma_{x^{*}},x^{*}\right)={\left(2\pi \right)}^{-k/2}{\left|\Sigma_{x^{*}}\right|}^{-1/2}\;\exp\left[-\frac{1}{2}{\left(x-x^{*}\right)}^{T}{\Sigma_{x^{*}}}^{-1}\left(x-x^{*}\right)\right].$$

Its log likelihood can be denoted as

(18) $$\ln p(x\left|\Sigma_{x^{*}},x^{*}\right)=-\frac{k}{2}\ln\left(2\pi \right)-\frac{1}{2}[\ln(|\Sigma_{x^{*}}\left|\;\right)+\underset{\left(a\right)\;\mathrm{Distance}\;\mathrm{metric}}{\underset{︸}{{\left(x-x^{*}\right)}^{T}\Sigma_{x^{*}}^{-1}\left(x-x^{*}\right)}}].$$

The Hessian matrix of the negative log likelihood (e.g., as in [33]) reduces to

(19) $$H_{k,l}\left(x^{*}\right)={\left.\;\frac{-\partial^{2}log\left(p\left(x\right)\right)}{\partial x_{k}\partial x_{l}}\right|}_{x=x^{*}}={\left(\Sigma_{x^{*}}^{-1}\right)}_{k,l},$$

which equals the observed Fisher Information matrix $I(x^{*})$ [34]. The relationship shown in Equation (19) provides the means for estimating a local covariance estimate at the point $x^{*}$ as the analytical solution of the Hessian matrix of the epistemic uncertainty, which can be computed analytically. Interestingly, the comparison of the epistemic uncertainty estimate U(x) (originating in Equation (6) to the log likelihood of a Gaussian PDF, as shown in Equation (18), reveals that both share the same characteristics of a distance metric. The respective similarities between Equation (6)(b) and Equation (18)(a) are the basis for the following approximation assumptions. In the case of our non-linear projection from inputs into the feature space, norms of hidden state vectors are assumed to be constant (as discussed previously; Section 3.1); in addition, the feature projection ϕ(x) can be considered as “distance preserving”, as it is a random projection. Therefore, we consider the local approximation,

(20) $$U(x^{*}+\delta )\underset{∼}{\propto }\ln p(x^{*}+\delta |\Sigma_{x^{*}},x^{*})$$

with $\Sigma_{x^{*}}=H_{U}^{-1}\left(x^{*}\right)$, the Hessian of the epistemic uncertainty U. In practice, iterative optimization can result in estimates $U(x^{*}+\delta )$ that are not symmetric or not positive-definite, due, e.g., to numerical inaccuracies. To increase the robustness of the numerical calculations, the results presented in the following are generated by estimating local covariance estimates according to $\Sigma_{x^{*}}={\left[\frac{1}{2}H_{U}\left(x^{*}\right)+\frac{1}{2}H_{U}^{\;T}\left(x^{*}\right))\right]}^{-1}$.

The Hessian matrix $H_{U}\left(x\right)$ of the epistemic uncertainty U at point x can be denoted in terms of the Jacobian matrix $J_{\nabla_{x}U}\left(x\right)$ of the gradient $\nabla_{x}U$, such that

(21) $$H_{U}\left(x\right)=J_{\nabla_{x}U}{\left(x\right)}^{T}={\left[\begin{array}{ccc} \frac{\partial \nabla_{x}U}{\partial x_{1}} & \cdots & \frac{\partial \nabla_{x}U}{\partial x_{n}} \end{array}\right]}^{\;T},$$

with

(22) $$\frac{\partial \nabla_{x}U}{\partial x_{j}}=2\frac{\partial }{\partial x_{j}}W_{inp}\left[S_{N}\phi \left(x\right)\right]\left(\phi \left(x\right)\left(1-\phi (x)\right)\right).$$

With substitutions g(x)=ϕ(x) for the inner function and $f\left(g\left(x\right)\right)=\frac{\partial }{\partial x_{j}}W_{inp}\left[S_{N}g\left(x\right)\right]\left(g(x)(1-g(x))\right)$ as the outer function, and under consideration of the symmetry of the Hessian matrix, we can denote

(23) $$\begin{array}{c} H_{U}\left(x\right)=2\left(J_{f\circ g}\right)\left(x\right)=2J_{f}\left(g\left(x\right)\right)\;J_{g}\left(x\right). \end{array}$$

The Jacobian $J_{g}\left(x\right)$ of the inner function *g* (i.e., random feature projection) is derived as

(24) $$J_{g}\left(x\right)=\mathrm{diag}\left(\phi \left(x\right)-\phi {\left(x\right)}^{2}\right)W_{inp},$$

and the elements of the Jacobian matrix $J_{f}\left(g\left(x\right)\right)$ of the outer function *f* are defined as

(25) $$\begin{array}{cc} \frac{\partial f_{i}\left(g\right)}{\partial g_{j}} & =\frac{\partial }{\partial g_{j}}W_{inp}\left[S_{N}g\right]\left(g(1-g)\right). \end{array}$$

Separation of *f* leads to expression

(26) $$\begin{array}{cc} \frac{\partial f_{i}\left(g\right)}{\partial g_{j}}= & W_{{inp}_{i,j}}S_{N_{j,j}}\cdot \left(2g_{j}-3g_{j}^{2}\right)\\ \; & +\sum_{k\neq j}W_{{inp}_{i,k}}S_{N_{k,j}}\cdot \left(g_{k}\left(1-g_{k}\right)\right)+W_{{inp}_{i,j}}\left(1-2g_{j}\right)\sum_{l\neq j}S_{N_{j,l}}g_{l}, \end{array}$$

and further details are provided in Appendix A. For efficient implementation, the Jacobian $J_{f}\left(g\left(x\right)\right)$ can be written in tensor notation, as detailed in Appendix B.

#### 3.6.2. Unlearning

The term *unlearning*, such as in Nguyen et al. [35], refers to successive updates of posterior estimates over model parameters W, such that it is as if a subset $D^{-}$ of the initial training set D was not considered for training. Such incremental updates are required, for example, in incremental learning scenarios, in cases where access or storage of the initial training data is not feasible. The posterior after unlearning can be expressed as

(27) $$p\left(W|D^{\subset }\right)=\frac{p\left(W|D\right)p\left(D^{-}|D^{\subset }\right)}{p(D^{-}|W)}\propto \frac{p(W|D)}{p(D^{-}|W)},$$

with $D^{\subset }=D\setminus D^{-}$, i.e., $D^{\subset }\cap D^{-}=⌀$. If we consider Bayesian linear regression, and given a conjugate Gaussian prior $N(W|m_{N},S_{N})$ on the weights W, as introduced in Equation (2), the exact solution, e.g., Rawat et al. [36], for the updated posterior distribution is given by

(28) $$\begin{array}{cc} m_{N}^{*} & =S_{N}^{*}\left(S_{N}^{-1}m_{N}+\beta {X_{\phi }^{-}}^{T}Y^{-}\right),\;\mathrm{with} \end{array}$$

(29) $$\begin{array}{cc} S_{N}^{*} & ={\left(S_{N}^{-1}-\beta {X_{\phi }^{-}}^{T}X_{\phi }^{-}\right)}^{-1}. \end{array}$$

For auto-association, as introduced in Section 3.5.1, targets Y and, thus, mean estimates $m_{N}$ do not exist. But an update of $S_{N}$ is sufficient for an update of the estimate of the epistemic uncertainty. Furthermore, a perfect removal of samples is not possible, and the assumption $D^{\subset }\cap D^{-}=⌀$ does not hold in practice. Violation of this condition can be caused by data noise or by attempted unlearning of untrained or generalized data samples. Therefore, we consider $D^{∼}$ as a set of sample candidates for deletion from D.

In a case where samples from $D^{∼}$ are sufficiently similar, i.e., an approximate subset of D, and $D^{\subset }\cap D^{∼}\approx ⌀$ holds, our empirical study shows that the approximate update, given samples $X_{\phi }^{∼}\in D^{∼}$,

(30) $$S_{N}\leftarrow {\left(S_{N}^{-1}-\eta^{-}\beta {X_{\phi }^{∼}}^{T}X_{\phi }^{∼}\right)}^{-1},$$

with $\eta^{-}≲1$ (e.g., $\eta^{-}=0.9$ in our experiments), is sufficient for reshaping the attractor basins appropriately, as shown in Section 4.4.

To overcome the stability issues of the analytically exact unlearning of training samples, we propose an iterative update procedure, as detailed by Algorithm A3 listed in Appendix D. The samples in $D^{∼}$ are weighted by an exponentially decaying function in relation to their epistemic uncertainty, i.e., samples with a high epistemic certainty are selected for unlearning, whereas uncertain samples are ignored.

### 3.7. Summary of Method Application

As detailed in Section 3.5 and Section 3.6, the proposed method demonstrates versatility in addressing a range of machine learning tasks. Depending on the specific application, the method operates in different modes. For outlier detection, the method requires only estimation of the epistemic uncertainty (U; Equation (6)) for a given input feature point. In contrast, tasks such as auto-completion and regression involve calculating the derivative of the epistemic uncertainty ($\nabla_{x}U$; Equation (10)) with respect to the input feature space, enabling minimization of uncertainty through iterative updates in the input feature space. At local estimates of minimum uncertainty given an input feature, local covariance approximation is based on the Hessian of the epistemic uncertainty ($H_{U}\left(x\right)$; Equation (21). Unlearning, on the other hand, necessitates adapting the model’s representation ($S_{N}$; Equation (30)) by updating how uncertain features are encoded within the hidden space. With regard to additional implementation, specific information on the representation of training data (Algorithm A1) and the estimation of epistemic uncertainty (Algorithm A2) is listed in Appendix C, example code is provided as Supplementary Material.

In the subsequent sections, we evaluate the model’s performance across these tasks and highlight the distinctive characteristics of its internal representations.

## 4. Experiments and Evaluation

### 4.1. Regression

**Figure 4:**
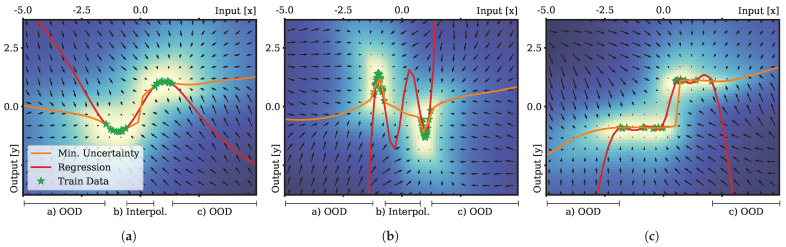
Results of the regression experiments. Regression was evaluated on three different datasets (**a**–**c**). The comparison between Bayes linear regression (red) and regression based on the minimization of the epistemic uncertainty (orange) is presented. Color shading of the background and the vector field indicates the epistemic uncertainty and its gradient field. Green stars depict training samples. A whitening transformation of the data samples (green) was performed as preprocessing to ensure zero mean and unit variance for each training dataset.

Our proposed method for function approximation presented in Section 3.5.2 differs significantly from more classical regression approaches. We stipulate that such regression, based on minimization of the epistemic uncertainty, can provide benefits, particularly in cases of strong generalization and extrapolation, unbalanced and multimodel input data distributions, and for approximation of noncontinuous functions. In cases of out-of-distribution (OOD) generalization, i.e., extrapolation to test samples far from the ones observed during training, overshooting of predictions due to overfitting are a common challenge for regression models. Therefore, we performed an evaluation of function approximation on three data distributions. In the first experiment, the training data were generated by sampling 20 values from a sine wave function for estimation of regression solutions. The inputs were drawn equally from two normal distributions with centers μsin∈{−π2,π2} and variances $\sigma_{\sin}^{2}\in \left\{0.5,0.5\right\}$. The resulting training data are shown in Figure 4a. For the second experiment, we increased the curvature (difficulty) of the function underlying the training data generation. In this case, we sampled 40 times from Gaussian probability density functions with $\sigma_{\mathrm{gauss}}^{2}=0.4$; the sampling inputs were drawn from two normal distributions with centers $\mu_{\mathrm{gauss}}\in \left\{-\pi ,\pi \right\}$ and variances $\sigma_{\mathrm{gauss}}\in \left\{0.4,0.4\right\}$. Where the input coordinates were drawn from the first distribution, the output was multiplied by −1 to generate a training dataset with antagonistic peaks, as shown in Figure 4b. In the third case, we sampled from a step function, which is usually difficult to represent with continuous function approximators. High regularization of the output weights was required, to avoid overfitting; however, low regularization was required, to represent a sharp step response. Classical regression approaches are restricted to a Pareto optimum between accuracy and generalization characteristics, due to additive loss terms, referred to as the *bias-variance dilemma* in the literature. In every case, a whitening transformation (preprocessing to ensure unit variance and zero mean) of the training data is performed in advance. We compare the standard Bayesian linear regression on a random projection of the input with our proposed approach. For evaluation of both methods, we used the same random projection with parameterization, as listed in Table 1.

Regression based on the proposed method was performed according to Section 3.5.2: starting from initial output state $y_{s=0}^{\left(n=0\right)}=0$, for sample *s* and optimization iteration *n* we successively set the input to values in the interval $x_{s}\in \left[-5,5\right]$. For each successive test input $x_{s+1}$, we set the respective initial value to $y_{s+1}^{\left(0\right)}\leftarrow y_{n}^{\left(N\right)}$ before minimization of the epistemic uncertainty with respect to $y_{s}^{n+1}$. Minimization of the epistemic uncertainty was performed by the efficient Broyden–Fletcher–Goldfarb–Shanno (BFGS; Fletcher [37]) optimization technique provided by the scientific *scipy* Python package [38] (version 1.14.1). For optimization, we specified precision threshold $1\times 10^{-3}$ and a maximum of $1\times 10^{3}$ optimization iterations.

Results:

The outcomes of our evaluation support our initial hypothesis: regression performed on the basis of minimization of epistemic uncertainty showed significantly less overshooting behavior in comparison to the standard regression approach. In all three cases, the standard ridge regression approach resulted in estimations far off the training samples in cases of out-of-distribution generalization. In particular, in cases with strong curvatures and gaps in the training distribution (Figure 4b) overfitting occurred even during interpolation between the first and second clusters of the training samples.

### 4.2. Novelty and Outlier Detection

We evaluated the proposed epistemic uncertainty estimator against common methods for outlier detection, such as the IForest, K-Nearest Neighbor estimates, PCA-based and Kernel PCA (KPCA)-based methods, and Gaussian mixture models (GMMs). For evaluation, we relied on the implementation and the datasets provided by the anomaly benchmarking software ADBench (version 0.1.11) [39]. We performed unsupervised outlier detection, i.e., we provided unlabeled samples and the ratio of outliers in the provided dataset to the models. Outlier detection was then based on a distance value provided by the respective method and a threshold operation. The threshold was determined for each model equally, as the $\left(1-d_{r}\right)$th percentile, which meant that we estimated the threshold at which the number of provided outlier samples rejected by the model matched the ratio $d_{r}$ provided by the datasets. We performed our analysis on a random subset of the provided datasets, and the results are listed in Table 2. The evaluation metric was the area under the receiver operating characteristic curve (AUCROC) value, a widely used metric in anomaly detection, the same metric referred to in the ADBench benchmark. All our results reflected the mean of 10 runs with the respective 0.95 percentile confidence interval. For the performance estimation of our proposed approach, we performed a grid search over the hyperparameters of the method, to determine the best-performing condition. Note that the competitive models we tested against in the ADBench likewise implemented an automatic hyperparameter estimation.

Results:

The evaluation shows that the performance of our proposed approach was in the range of the common methods for outlier detection. We could identify multiple datasets for which the epistemic uncertainty estimator performed best under the given conditions. As the authors Han et al. [39] mention in their work, the performance of the models for outlier detection is dataset-dependent, and a model search is usually performed for specific use cases.

### 4.3. Local Covariance Approximation

**Figure 5:**
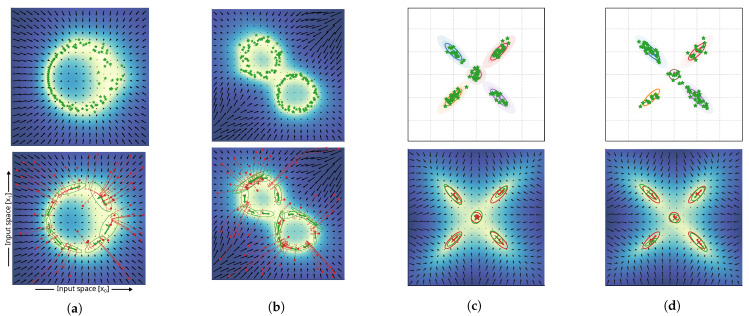
Experiments regarding the assessment of the quality of local approximation of Gaussian distributions. For each experiment, one sub–figure, (**a**–**d**), shows the distribution of the training data (top) and the results of the approximated covariance matrices (bottom). The lower panels of (**a**,**b**) indicate random samples initial states (red circles) and optimization paths for mean estimation (dashed red line). Experiments (**c**,**d**) used data sampled from a Gaussian mixture with five clusters and the ground truth covariance indicated as colored ovals (shown in the top panels). The lower panels of (**c**,**d**) indicate estimates of fitting a Gaussian mixture model (red) and estimates generated by local covariance approximation of the epistemic uncertainty (green). The experiment shown in (**d**) introduces different weightings of the Gaussian distributions for sampling; weighting factors were x1 (orange and brown), x2 (red), x3 (blue), and x4 (purple). Weighing is also indicated by the number of samples drawn from each distribution (green stars). Color shading of the background relates to the epistemic uncertainty values for each coordinate.

Cluster Discovery:

The method for covariance estimation, as introduced in Section 3.6.1, is a local approximation and assumes a normally distributed uncertainty landscape at converged point-wise minima of the epistemic uncertainty. Obviously, there exist also distributions that violate these assumptions. To evaluate the feasibility of local covariance estimates, we tested our estimates on a set of distributions, as shown in Figure 1 and Figure 5. Starting from random initial states in the input space, we minimized the epistemic uncertainty according to Equation (16); finally, we performed a local Gaussian approximation by estimating the covariance, Equation (20), by use of the assumption introduced in Equation (19). The plots show the optimization trajectories (red lines) of the gradient descent and the final covariance estimates (visualized as red ellipses) with centers (marked by symbol x in red).

**Figure 6:**
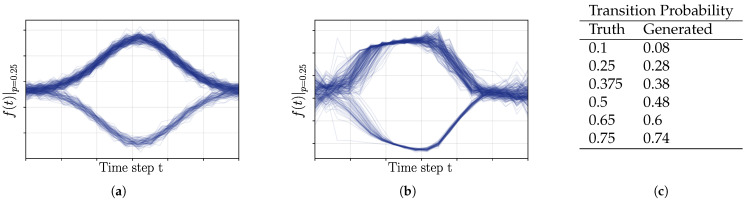
Experiments on probabilistic trajectory generation: (**a**) Training data used in the case of a switching probability of 0.25. One of two possible trajectories is generated—upper path or lower path; time steps are depicted on the horizontal axis. (**b**) The experimental results of the proposed method, in the case of a switching probability of 0.25, were used during the sampling of the training data. (**c**) List of target and mean reconstructed switching probabilities of 250 trajectory generation trials each.

Probabilistic Trajectory Generation:

Given the results of an accurate 2D distribution recovery, shown in the previous paragraph, we further challenged the proposed method with the representation of time series trajectories with probabilistic branching. The trajectories and branching probabilities tested are shown in Figure 6a and Figure 6c, respectively. For this experiment, each observation sample used for training included the current state, the future state, and the current time, i.e., we were operating on three-dimensional observation vectors. For trajectory generation, we sampled the next state vector (the output) from the estimated distribution, as specified in Section 3.6.1. Given a next time stamp and a current state (last output estimate), the epistemic uncertainty was minimized, with respect to the next state output. The final output estimate was then calculated through sampling from a local Gaussian approximation based on the covariance estimation according to Equation (20), as performed in the previous experiment.

Results:

The results in Figure 5 show that the characteristics of the training distributions provided (the samples indicated by green star marks) were represented by the estimated Gaussian distributions of the epistemic uncertainty. Even in cases of continuous circular distributions, local covariance estimation resulted in meaningful Gaussian approximations. In case of varying variance of the sampling process in the dataset generation (e.g., variance of sampling increased towards the right part of the data distribution in Figure 5a; variance of the top-left circular distribution was larger compared to the lower-right one in Figure 5b), it can be seen that the respective variance of the Gaussian approximations resembled the characteristics of the variance of the training data distribution. The application of the method on a dataset sampled from a Gaussian mixture model with equal weights of all five Gaussian distributions (Figure 5c) shows the successful discovery of the underlying ground truth distribution (top panel). It can be seen that the estimated local covariances show similarities to the ones estimated through fitting a GMM. The KL divergence between GMM and ground truth distribution reached 0.14±0.002 with a 95% confidence interval. The KL divergence between the estimated Gaussians through local covariance approximation of the epistemic uncertainty was found to be 0.22±0.006. Both were estimated on 20 experiment repetitions. The experiment shown in Figure 5d explored the limitations of local approximation of probability distributions, as it introduced uneven weighting between the sample distributions of the ground truth. Our proposed method cannot represent the global relationship between local probabilistic representations and cannot estimate the weighting factors for each estimated distribution. As a result, as shown in the lower panel of Figure 5d, the weighting of the components in the sample GMM resulted in differences in the estimated size of the local covariance approximations. The cluster sampled from the distribution with the lowest weight (orange) resulted in the smallest distribution, while clusters with increasing weights of distributions for sampling were estimated with increasing size. However, the orientation of the estimated clusters still resembled the correct orientation of the ground truth. In this case, the approximation of a GMM using the expectation maximization (EM) algorithm was beneficial and resulted in a KL divergence of 0.18±0.023, and the estimated distribution based on the local covariance estimations reached a KL-divergence of 0.51±0.014.

### 4.4. Unlearning in Case of Noise

**Figure 7:**
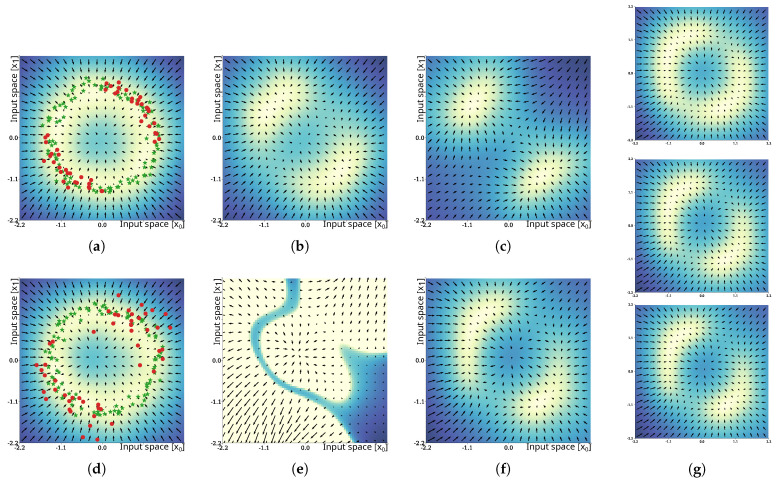
Results of the unlearning experiment. The results for unlearning of samples drawn from the same distribution of the training data are shown in the top row. The bottom row shows the results of unlearning samples drawn from a distribution with increased variance. The training and samples to be unlearned are shown in the first column, (**a**,**d**). The results of unlearning using the naïve update method are shown in the second column, (**b**,**e**). The results of the proposed iterative unlearning procedure after 20 iterations can be found in the third column, (**c**,**f**). Panel (**g**) depicts intermediate results at 5, 10, and 15 iterations for the second experimental condition. Color shading of the background and the vector field indicate the epistemic uncertainty and its gradient field.

For the evaluation of the unlearning capabilities of the proposed method, we refer to a circular distribution of training samples, as shown in Figure 7a,d, similar to the distributions we used in our previous experiments. Training samples $v_{\mathrm{tr}}\in D$ are indicated by green star-shaped marks. Unlearning was performed under two conditions: firstly, we attempted to unlearn samples from the same distribution that we used to sample the training data (Figure 7a; results in Figure 7b,c); secondly, we increased the sample variance by factor x2 (Figure 7d; results in Figure 7e,f) to increase the task difficulty. Samples for removal $v_{\mathrm{rm}}\in D^{∼}$ are visualized by red circles. For each of the two experimental conditions, we evaluated naïve unlearning according to Equation (30) and incremental unlearning according to our proposed method as specified in Appendix D.

Results:

In the cases where unlearning was performed with samples $x_{\mathrm{rm}}$ drawn from the same distribution as used for the training samples $x_{\mathrm{tr}}$, unlearning was successful, using the naïve approach with update rate $\eta^{-}=0.9$, as shown in Figure 7b. The epistemic uncertainty in the 2nd and 4th quadrant was considerably increased, and successive trials of maximization of the epistemic uncertainty would avoid unlearned parts of the distribution. Iterative unlearning resulted in similar, slightly smaller areas of low uncertainty (Figure 7c). Both methods can be considered to have been successful in these trials. In the cases where unlearning was performed with samples $x_{\mathrm{rm}}$ drawn from distributions with increased variance, the results changed drastically. Under this condition, unlearning with the naïve approach failed and resulted in a loss in representation of the training data, as shown in Figure 7e. As discussed in Section 3.6.2, unlearning of dissimilar samples violates assumption $D^{\subset }\cap D^{∼}\approx ⌀$, which can result in negative values of $S_{N}$ and can break the necessary symmetry of $S_{N}$. Simply decreasing the update rate $\eta^{-}$ is not sufficient, as it reduces the effect of increasing the epistemic uncertainty and may cause local minima to remain in parts of the distribution that were intended to be unlearned. In the cases where unlearning was performed using the proposed iterative approach, the solutions converged and resulted in an epistemic uncertainty distribution, as shown in Figure 7f. The shown result depicts the 20th update iteration with parameterization $\eta^{-}=0.5$ and ρ=3. Unlearning can be considered successful in this case. Further intermediate update steps at iterations #5 (top), #10 (middle), and #15 (bottom) are shown in Figure 7g and indicate that convergence of unlearning occurs at ∼10 iterations.

## 5. Discussion and Conclusions

The presented work uses epistemic uncertainty as a data model and tackles classical learning problems from a new perspective by utilizing the epistemic uncertainty gradient. Typically, learning is considered as the representation (based on error minimization) of training targets and model outputs. The quality of the learning methods is then interpreted as the ability to interpolate and extrapolate on the basis of the internalized training samples. In our proposed approach, learning is solely based on the representation of the training data in terms of their potential predictive distribution variance and does not rely on explicit calculation of output targets.

We have demonstrated that classical learning problems such as outlier/anomaly detection, auto-completion as in associative memories, and regression tasks can be implemented by using the epistemic gradient. In addition, we propose and evaluate approaches for local covariance estimates of the learned data distributions and unlearning of data that are robust against noise and can deal with relatively small overlap of the data, to unlearn with the original distribution.

The presented theoretical analysis leads to a geometric interpretation of epistemic uncertainty gradient that differs fundamentally from the ones found in classical learning approaches. Solutions that are found through minimization of the epistemic uncertainty are not formed by attractors based on the training data. Instead, the gradient is based on a representation of unfavorable solutions that can be considered to act as repellers pushing away from the “unknown” and, therefore, implicitly approaching the “known”, i.e., approaching the modeled data distribution as given through the example data. As discussed in this work, the feature projection from the input space onto an invariant manifold in the feature space is crucial as it enables non-trivial dynamics toward the previously observed data distribution through an ensemble of “simple” repelling forces in the feature space during minimization of the epistemic uncertainty. One key factor of our work that enables this complex interaction is the random projection, which can be considered distance-preserving and unbiased, and is, therefore, ideal for preserving information in the hidden space. The described mechanism and its geometrical interpretation are, to the best of our knowledge, a new concept in data modeling. 

On a more abstract and fundamental level, the proposed approach introduces the question of what are the differences between learning data (attractor dynamics) and avoiding improbable solutions (repelling mechanisms) and how they play out in practice.

While we cannot give a comprehensive answer yet, we have addressed this question, at least partially, through comparing the solutions of classical ridge regression and the epistemic uncertainty gradient for simple function approximation, where overfitting is a common problem. Our findings suggest that the epistemic uncertainty approach may be advantageous when strong generalization and extrapolation are required, where the epistemic gradient fields appear to be much more implicitly regularized. The insights gained from our study are highly relevant for robust learning, e.g., construction of efficient world models that require strong generalization as the available data are usually sparse, ambiguous, and noisy. More work is needed, to further characterize these differences and the properties of the epistemic uncertainty gradient field.

### Outlook

An important practical aspect of this potential work will be the application of the presented approach to real-world datasets, with a specific emphasis on scalability to large-scale datasets, noise and imbalanced data, and sparsity of information. Theoretical investigations could explore if further model architectures, such as multi-layered models, can implement the presented mechanism for data representation. Potential related studies could address topics of hierarchical attractor networks, and clarify if, in such cases, local multi-layer learning without gradient propagation is feasible.

Additionally, we are interested in exploring synergies between our method and approaches that explicitly learn attractor representations of training data, as in Reinhart and Steil [40], as well as those proposed more recently in the deep learning community, such as diffusion models and denoising auto-encoders [41]. We speculate that the presented approach could lead to potential advancements in representation learning.   

## Figures and Tables

**Table 1:** Model parameterization of regression experiments.

Model Parameterization			
	**Dims**	α	β
Exp. 1	80	$1\times 10^{-2}$	$1\times 10^{+1}$
Exp. 2	120	$1\times 10^{-3}$	$1\times 10^{+4}$
Exp. 3	200	$1\times 10^{-4}$	$2\times 10^{+1}$

**Table 2:** Evaluation of our proposed approach against common approaches used for anomaly detection. The datasets and the implementation of the model are based on the work of Han et al. [39]. Evaluation was performed in an unsupervised fashion, without knowledge of the class label of each sample. Only the ratio of out-of-class samples in the dataset was known a priori. The evaluation metric shown is the AUCROC score and its confidence interval (95%-CI). Each row marks the best performing method in bold font.

	IForest	KNN	PCA	KPCA	GMM	Ours
**Cardio**	0.94±0.01	0.77±0.00	0.96±0.00	0.73±0.00	0.92±0.00	0.89±0.01
**BreastW**	0.99±0.00	0.98±0.00	0.95±0.00	0.98±0.00	0.98±0.00	0.97±0.00
**Glass**	0.62±0.04	0.76±0.00	0.34±0.00	0.76±0.00	0.49±0.00	0.92±0.01
**Speech**	0.52±0.02	0.53±0.00	0.52±0.00	0.60±0.00	0.56±0.00	0.57±0.02
**Landsat**	0.49±0.01	0.59±0.00	0.36±0.00	0.56±0.00	0.46±0.00	0.73±0.01
**Hepatitis**	0.77±0.03	0.84±0.00	0.79±0.00	0.86±0.00	0.85±0.00	0.75±0.02
**Stamps**	0.90±0.01	0.84±0.00	0.91±0.00	0.80±0.00	0.87±0.00	0.91±0.02
**Thyroid**	0.98±0.00	0.96±0.00	0.95±0.00	0.96±0.00	0.93±0.00	0.94±0.00
**Vertebral**	0.46±0.03	0.39±0.00	0.44±0.00	0.40±0.00	0.44±0.00	0.64±0.01
**Yeast**	0.38±0.01	0.41±0.00	0.43±0.00	0.38±0.00	0.40±0.00	0.54±0.01

## Data Availability

The original contributions presented in this study are included in the article/Supplementary Material. Further inquiries can be directed to the corresponding author.

## Supplementary Materials

The following supporting information can be downloaded at https://www.mdpi.com/article/10.3390/e27020144/s1, Example Code: EpiGrad_model.html; EpiGrad_unlearn.html; EpiGrad_interactive.html.

## Appendix A. Implementation—Specific Details: Derivation of Epistemic Uncertainty

Separation of *f* in Equation (25) leads to expressions

(A1) $$\begin{array}{cc} \frac{\partial f_{i}\left(g\right)}{\partial g_{j}}= & \frac{\partial }{\partial g_{j}}\sum_{k}W_{{inp}_{i,k}}\sum_{l}S_{N_{k,l}}g_{l}\cdot \left(g_{k}\left(1-g_{k}\right)\right)\\ = & \frac{\partial }{\partial g_{j}}\sum_{k,l}W_{{inp}_{i,k}}S_{N_{k,l}}g_{l}\cdot \left(g_{k}\left(1-g_{k}\right)\right)\\ = & \frac{\partial }{\partial g_{j}}W_{{inp}_{i,j}}S_{N_{j,j}}g_{j}\cdot \left(g_{j}\left(1-g_{j}\right)\right)\\ + & \frac{\partial }{\partial g_{j}}\sum_{k\neq j,l=j}W_{{inp}_{i,k}}S_{N_{k,j}}g_{j}\cdot \left(g_{k}\left(1-g_{k}\right)\right)\\ + & \frac{\partial }{\partial g_{j}}\sum_{k=j,l\neq j}W_{{inp}_{i,j}}S_{N_{j,l}}g_{l}\cdot \left(g_{j}\left(1-g_{j}\right)\right)\\ + & \frac{\partial }{\partial g_{j}}\sum_{k\neq j,l\neq j}\underset{=0}{\underset{︸}{W_{{inp}_{i,k}}S_{N_{k,l}}g_{l}\cdot \left(g_{k}\left(1-g_{k}\right)\right))}}\\ = & W_{{inp}_{i,j}}S_{N_{j,j}}\cdot \left(2g_{j}-3g_{j}^{2}\right)\\ + & \sum_{k\neq j}W_{{inp}_{i,k}}S_{N_{k,j}}\cdot \left(g_{k}\left(1-g_{k}\right)\right)\\ \; & +W_{{inp}_{i,j}}\left(1-2g_{j}\right)\sum_{l\neq j}S_{N_{j,l}}g_{l}. \end{array}$$

## Appendix B. Implementation—Specific Details: The Hessian of the Epistemic Uncertainty

For efficient implementation, the Jacobian $J_{f}\left(g\left(v\right)\right)$ of the outer function *f* can be written in matrix notation as

(A2) $$\begin{array}{cc} J_{f}\left(g\left(v\right)\right)= & W_{inp}\circ \left[\mathrm{diag}\left(S_{N}\right)\left(2\phi \left(v\right)-3\phi {\left(v\right)}^{2}\right)\right]\\ + & \left[W_{inp}\circ \;\left(\phi \left(v\right)-\phi {\left(v\right)}^{2}\right)\right]S_{N}\\ - & \left[W_{inp}\circ \;\mathrm{diag}\left(S_{N}\right)\right]\circ \;\left(\phi \left(v\right)-\phi {\left(v\right)}^{2}\right)\\ + & \left[\left(S_{N}\phi \left(v\right)\right)\left(1-2\phi \left(v\right)\right)\right]W_{inp}\\ - & \left[\left(\mathrm{diag}\left(S_{N}\right)\phi \left(v\right)\right)\left(1-2\phi \left(v\right)\right)\right]W_{inp} \end{array}$$

Note that function diag(·) returns a diagonal matrix in cases where a vector is given as an argument and returns the vector of the diagonal elements in cases of a matrix. Furthermore, the operator ∘ denotes the Hadamard product and performs an expansion of vectors into matrices in cases where the product is calculated between a vector and a matrix.

## Appendix C. Implementation—Specific Details: Model Details

**Algorithm A1:** Representation of training set D, i.e., estimation of $S_{N}$, given hyperparameter α and β.

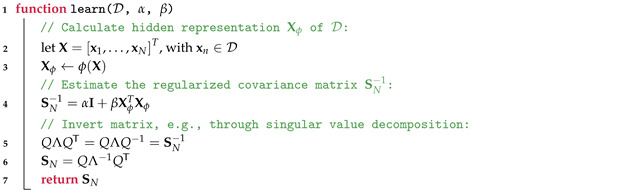

**Algorithm A2:** Query learned data distribution represented in $S_{N}$ on new data sample x. Perform *N* minimization steps of the epistemic uncertainty before returning final estimate $x^{\left(N\right)}$ and the epistemic uncertainty $U(x^{\left(N\right)})$.

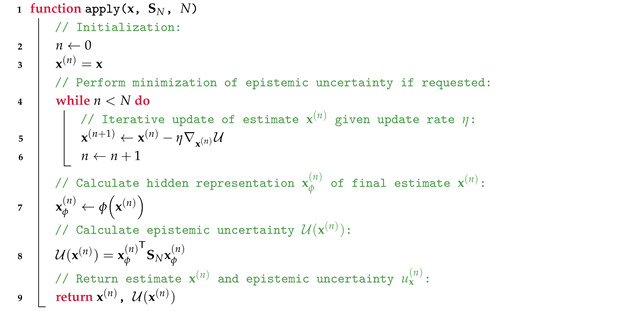

## Appendix D. Implementation—Specific Details: Iterative Approach to Unlearning

**Algorithm A3:** Iterative unlearning procedure.
