# Supplementary material for: The Epistemic Uncertainty Gradient in Spaces of Random Projections
Source: Entropy (Basel). 2025 Feb 1;27(2):144. doi: 10.3390/e27020144 (PMC11854594; doi:10.3390/e27020144)
Supplement: Supplementary file 1 [file entropy-27-00144-s001.zip › EpiGrad_interactive.html]

Paper Supplement: The Epistemic Uncertainty Gradient in Spaces of Random Projections


- Recalculate All
- Open Paper as PDF
- Research Page
